# TDP-43 related amyotrophic lateral sclerosis-frontotemporal dementia and links to the DNA damage response: a systematic review and narrative synthesis

**DOI:** 10.3389/fnmol.2026.1671909

**Published:** 2026-03-17

**Authors:** Seham Almalki, Mohamed Salama, Matthew J. Taylor, Zubair Ahmed, Richard I. Tuxworth

**Affiliations:** 1 Department of Cancer and Genomic Sciences, School of Medical Sciences, College of Medicine and Health, University of Birmingham, Birmingham, United Kingdom; 2 Department of Biotechnology, Faculty of Science, Taif University, Taif, Saudi Arabia; 3 Department of Inflammation and Ageing, School of Infection, Inflammation and Immunology, University of Birmingham, Birmingham, United Kingdom; 4 Birmingham Centre for Neurogenetics, University of Birmingham, Birmingham, United Kingdom; 5 University Hospitals Birmingham NHS Foundation Trust, Birmingham, United Kingdom

**Keywords:** ALS, ALS-FTD, DDR, DNA damage, DNA repair, FTD, TDP-43

## Abstract

Mislocalization and aggregation of the DNA/RNA binding protein, TDP-43, is seen in most cases of amyotrophic lateral sclerosis-frontotemporal dementia (ALS-FTD). Accumulating DNA damage in neurons is also a common feature of ALS-FTD. TDP-43 has several characterized roles in the regulation of the DNA damage response (DDR). This review systematically explored the relationship between TDP-43, DNA damage and the DNA damage response in various models of ALS-FTD, facilitating comparison of findings between studies using similar models. Twelve peer-reviewed papers, covering eight TDP-43 mutations out of nearly 40, were reviewed and five experimental models included: cell lines, patient-derived iPS cells, organoids, and rodent models, plus post-mortem cortex and spinal cord tissue from ALS-FTD patients. Across the studies and models, depletion of TDP-43 or ALS-linked mutations consistently increased genomic instability. Q331K-expressing cells showed a 2-3-fold reduction in DNA repair activity and a 4-6-fold increase in DDR activation, while *TDP-43*-depleted cells showed a 20-fold rise in double strand breaks. TDP-43 normally binds to damaged chromatin, participates in early DDR signaling and scaffolds core DNA damage repair factors, including Ku70, XRCC4 and DNA ligase 4. This systematic review and narrative synthesis sheds light on mechanisms that explain how TDP-43 dysfunction impairs genome maintenance. When TDP-43 is mislocalized, mutated or aggregated, these interactions are disrupted, resulting in impaired DNA repair. DNA damage is also caused by increasing R-loops, dysregulation of mismatch repair gene transcription, and sequestering of repair proteins into cytoplasmic inclusions. Upstream DNA damage can further drive TDP-43 mislocalisation, creating a feed-forward loop. Given the ubiquity of TDP-43 pathology across neurodegenerative diseases, targeting the DDR mechanisms affected by TDP-43 may offer new therapeutic opportunities.

## 1 Introduction

Amyotrophic lateral sclerosis (ALS) and frontotemporal dementia (FTD) form a spectrum of neurodegenerative diseases with overlapping clinical and molecular characteristics (ALS-FTD). ALS is marked by the gradual deterioration of motor neurons resulting in muscle weakness. ALS classically presents first with weakness of the lower motor neurons with later degeneration of the upper motor neurons that leads eventually to respiratory failure. ALS is an aggressive form of neurodegeneration with an average survival time of 3 to 5 years following diagnosis (Eshima et al., 2023; Tzeplaeff et al., 2023). Frontotemporal dementia is characterized by changes in behavior and difficulties with language, stemming from the atrophy of the frontal and temporal lobes (Neumann et al., 2009; Tzeplaeff et al., 2023). Neuropathologically, TDP-43 cytoplasmic aggregates are observed in 90% of ALS cases and 50% of FTD cases (Jo et al., 2020), highlighting TDP-43 proteinopathy as a characteristic of ALS-FTD spectrum disorders.

TDP-43, encoded by the *TARDBP* gene, is a predominantly nuclear protein that plays crucial roles in RNA processing, including splicing regulation, mRNA stability maintenance, and transport (Koike, 2024). The protein contains an N-terminal dimerization domain that also contains the nuclear localization sequence (NLS), with two central RNA recognition motifs essential for the role of TDP-43 as a splicing regulator. The unstructured C-terminal mediates binding with other proteins and is considered central to the mislocalization and aggregation of TDP-43 to the cytoplasm, which compromises its functional capacity, resulting in genomic instability and heightened vulnerability to neurodegenerative processes (Dang, et al., 2025).

Neurons are thought to be more vulnerable to DNA damage due to their high metabolic activity and their limited capacity to repair such damage, coupled with their longevity. Post-mitotic neurons experience primarily three forms of DNA damage: (a) oxidative lesions, which result from reactive oxygen or nitrogen species and lead to base modifications and single-strand breaks (SSB); (b) double-strand breaks (DSB), considered to be the most harmful type of DNA damage and which occur due to failed SSB repair or oxidative stress; and (c) the activation of transposable elements, linked to the loss of TDP-43 function in specific ALS subtypes (Martin, 2008; Chatterjee and Walker, 2017; Eshima et al., 2023). In post-mitotic cells, homologous recombination (HR) is inactive; consequently, these cells rely on error-prone non-homologous end-joining (NHEJ) and base excision repair (BER) to address DSB and oxidative damage, respectively (Martin, 2008; Chatterjee and Walker, 2017). TDP-43 also appears to regulate the splicing and transcription of two genes involved in the mismatch repair pathway (MMR): MLH1 and MSH6. This suggests a broader role for TDP-43 in the DNA damage response (DDR) that extends beyond conventional pathways (Provasek et al., 2025).

To date, three pharmaceutical treatments for ALS have received FDA approval: Riluzole, Edaravone, and Sodium phenylbutyrate/Taurursodiol. These treatments primarily function to slow disease progression and can slightly extend patient survival (Tzeplaeff et al., 2023). In contrast, FTD currently lacks any approved treatment options. Certain genetic therapies have been designed to address particular familial ALS conditions. For instance, the antisense oligonucleotide, Tofersen, targets SOD1 mRNA, triggering its degradation and consequently reducing production of the toxic SOD1 protein (Tzeplaeff et al., 2023). However, SOD1-associated ALS is only a tiny fraction of ALS cases, which leaves most patients with no effective treatments. An alternative therapeutic approach that might be more generalizable for ALS-FTD patients is to target the genomic instability and downstream effects associated with TDP-43. To aid understanding of how TDP-43 dysfunction leads to genomic instability and to highlight potential therapeutic targets, this review systematically explores the experimental findings from both *in vitro* and *in vivo* pre-clinical research to evaluate the involvement of TDP-43 in DNA damage and repair mechanisms in models of ALS-FTD.

This review follows the same methodology as two similar reviews that focused on connections between DNA damage, the DNA damage response and two other genes associated with familial ALS-FTD that have been studied using a wide variety of pre-clinical models: C9orf72 and FUS (Almalki et al., 2025a; Almalki et al., 2025b). When viewed together, the conclusions reached provide insight into common pathophysiological mechanisms at play across a range of disorders of the ALS-FTD spectrum.

## 2 Methods

### 2.1 Search strategy

**Figure 1:**
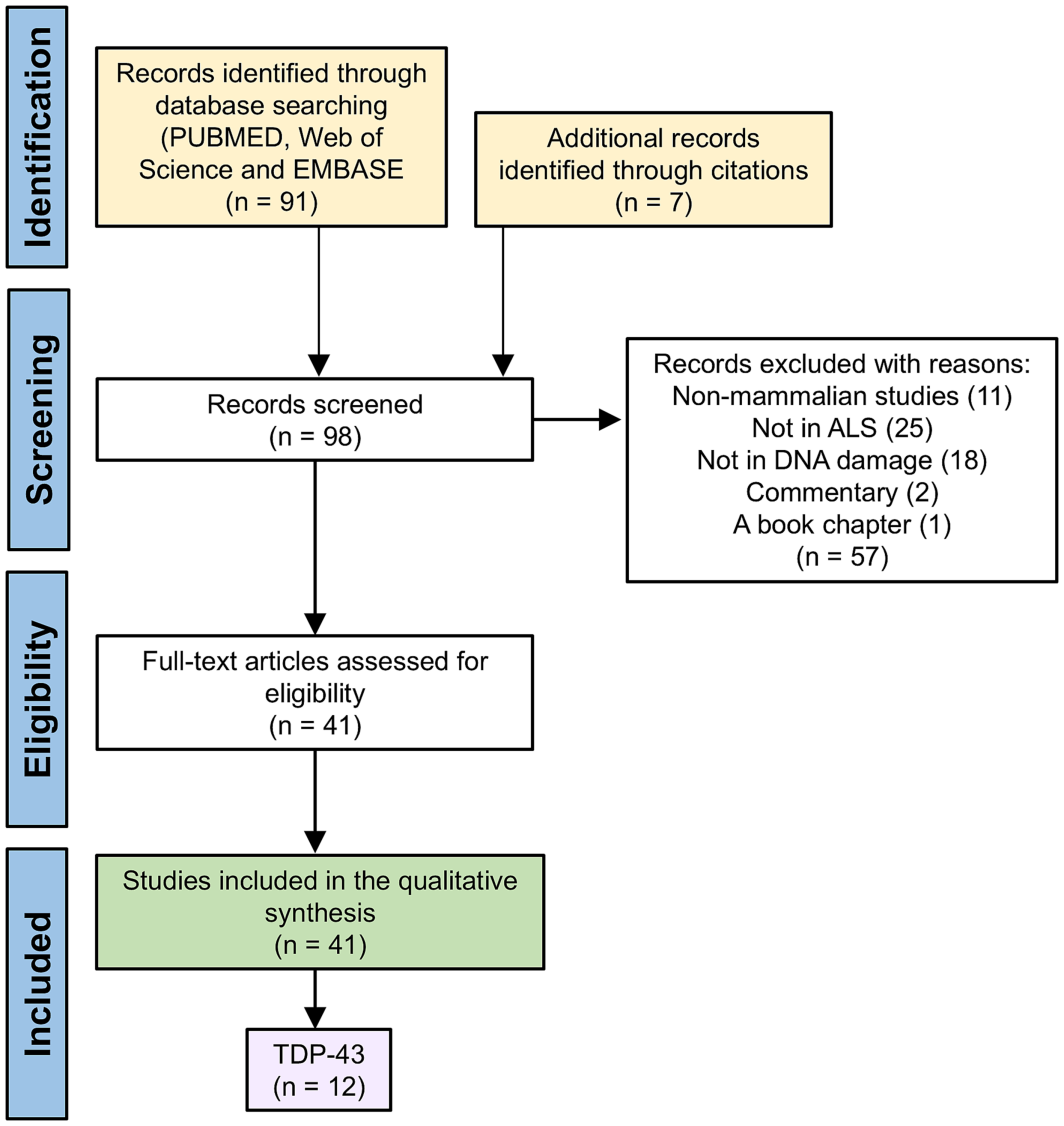
The PRISMA flow diagram indicating inclusion and exclusion criteria used in the systematic review.

This systematic review and narrative synthesis forms part of a larger review of the association of ALS-FTD and DNA damage in the nervous system that has been written as three related articles. The methodology used for the review is described in detail in the first article of the three that focuses on ALS-FTD associated with C9orf72 expansions (Almalki et al., 2025a). In brief, our literature search used the Boolean terms ‘amyotrophic lateral sclerosis’ OR ‘ALS’ AND ‘DNA’ AND ‘double strand breaks’ across PubMed, EMBASE and Web of Science databases for peer-reviewed primary research articles, in English and published to February 2025. This search yielded 91 publications after removing of duplicates. Of these, 41 articles were retained for full-text screening and only the 12 (Provasek et al., 2025; Gianini et al., 2020; Guerrero et al., 2019; Fang et al., 2023; Mitra et al., 2019; Nogami et al., 2022; Mitra et al., 2025; Lee and Rio, 2024; Konopka et al., 2020; Tamaki et al., 2023; Marques et al., 2024; Pal et al., 2021) that specifically focused on TDP-43 proteinopathy or dysfunction and its association with DNA damage, the DNA damage response (DDR), and DNA repair pathways were included for analysis (Figure 1).

## 3 Results

### 3.1 Basic information of included studies

The study selection and characteristics are detailed in the accompanying systematic review (Almalki et al., 2025a).

### 3.2 Risk of bias assessment results

**Figure 2:**
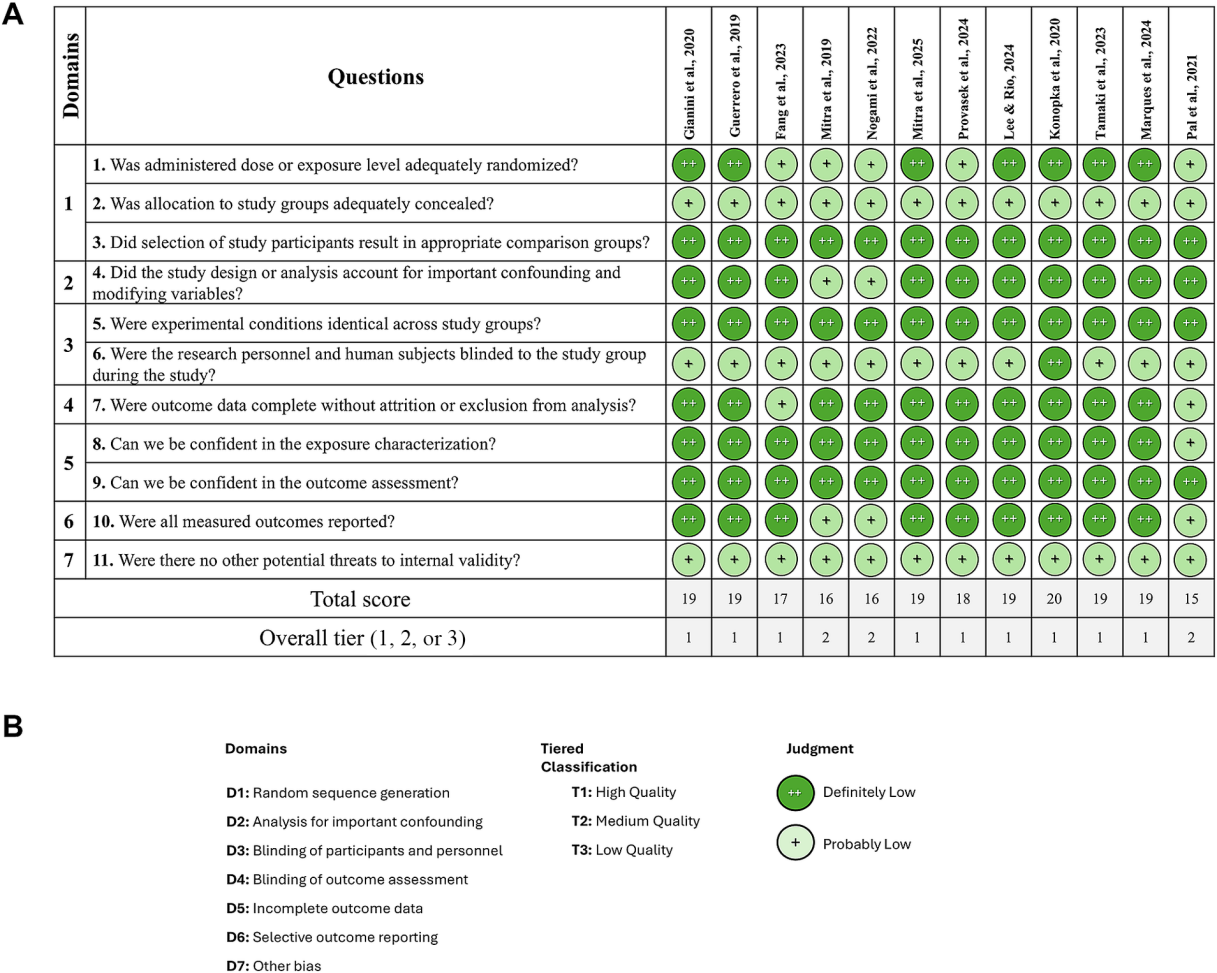
The OHAT tool for rating the risk of bias in *in vitro* studies. **(A)** Risk of bias assessment across included studies. **(B)** Risk of bias domains and classification criteria.

Each of the 12 studies included *in vitro* results. To assess Risk of Bias (RoB) in these studies we used the Office of Health Assessment and Translation (Ohat, 2019; Figure 2). The tool covers seven domains. Overall, 9 out of 12 studies (Provasek et al., 2025; Fang et al., 2023; Gianini et al., 2020; Guerrero et al., 2019; Marques et al, 2024; Mitra et al., 2025; Lee and Rio, 2024; Konopka et al., 2020; Tamaki et al., 2023) were classified as Tier 1, indicating a low RoB. In contrast, three studies (Nogami et al, 2022; Mitra et al., 2019; Pal et al, 2021) were identified as having moderate RoB and were classified as Tier 2. This suggests generally strong methodological rigor across the studies. Domains 5 (incomplete outcome data) consistently showed high confidence across all studies, with nearly all cases rated as “++.”

**Figure 3:**
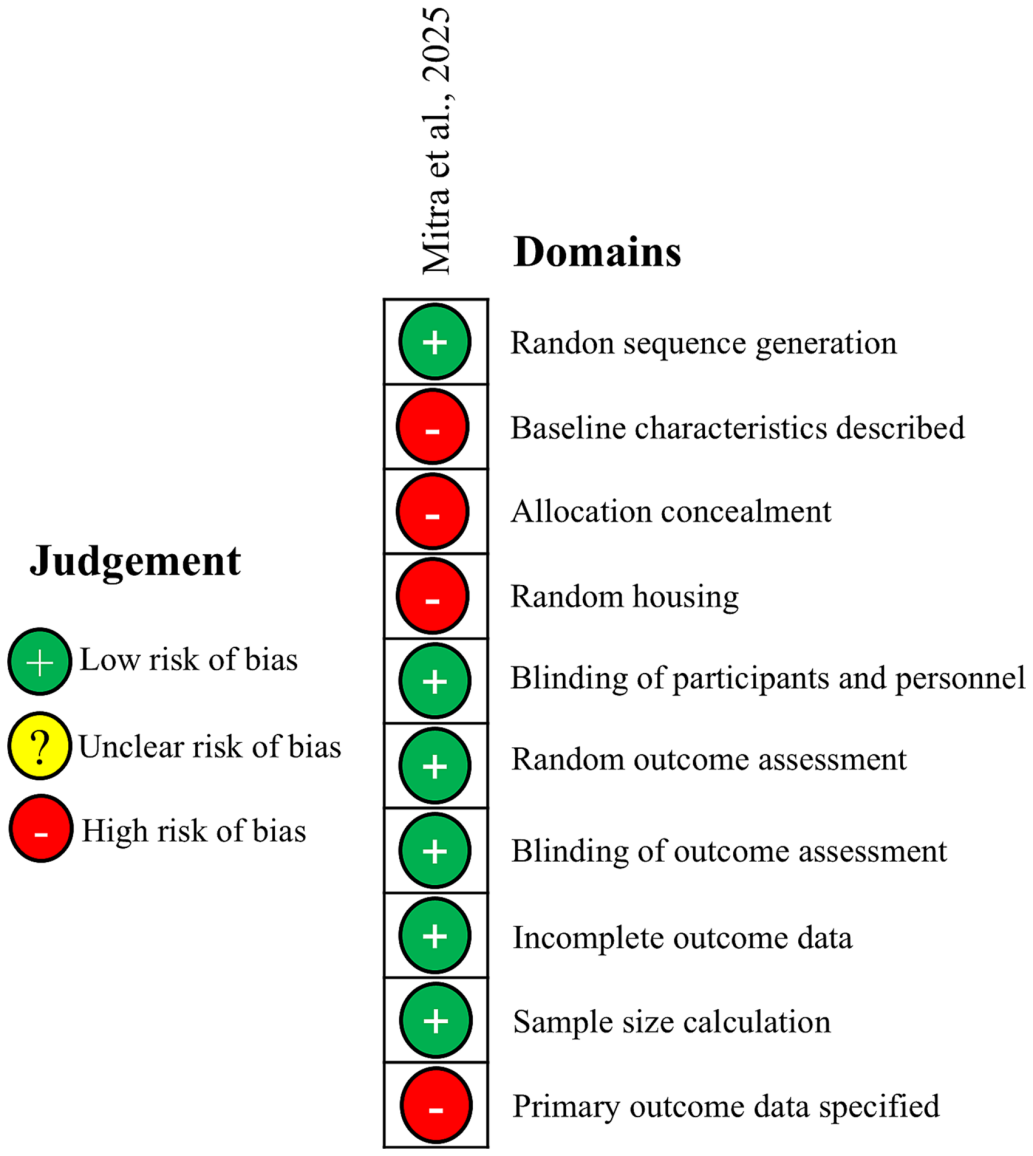
SYRCLE risk of bias assessment of the solo *in vivo* study.

Only one study presented vertebrate *in vivo* results (Mitra et al, 2025). For this study we employed the SYRCLE tool (Hooijmans et al, 2014) to assess RoB (Figure 3). Although this demonstrates low RoB in six critical domains, including the blinding of participants and outcome assessment, it identifies four domains with high RoB: baseline characteristics, random housing, allocation concealment and specification of primary outcome data.

In summary, while the *in vitro* studies exhibited overall low risk of bias, the *in vivo* study showed a high risk of bias in some domains.

### 3.3 Results of studies

The systematic review and narrative synthesis identified 12 publications that investigated the role of DNA damage, the DDR, and DNA repair mechanisms in TDP-43 proteinopathy in both neuronal and non-neuronal models. Five experimental systems were used to establish TDP-43 models, including immortalized cell lines of either human or rodent origin (Fang et al., 2023; Gianini et al., 2020; Guerrero et al., 2019; Konopka et al., 2020; Lee and Rio, 2024; Mitra et al., 2019; Mitra et al., 2025; Nogami et al., 2022; Provasek et al., 2025), organoids (Tamaki et al., 2023), induced pluripotent stem cells (iPSCs) derived from ALS cases (Fang et al., 2023; Gianini et al., 2020; Konopka et al., 2020; Marques et al., 2024; Mitra et al., 2019; Pal et al., 2021; Provasek et al., 2025), rodent tissues (Konopka et al., 2020; Mitra et al., 2025; Provasek et al., 2025), and postmortem tissues from ALS patients (Fang et al., 2023; Guerrero et al., 2019; Marques et al., 2024; Mitra et al., 2019; Provasek et al., 2025). The 12 studies were published from 2019 to 2025. Seven studies were carried out in the USA (Fang et al., 2023; Guerrero et al., 2019; Lee and Rio, 2024; Marques et al., 2024; Mitra et al., 2019; Mitra et al., 2025; Provasek et al., 2025) and the remaining five were in Italy/Spain (Gianini et al, 2020), Japan (Nogami et al, 2022), Australia (Konopka et al., 2020), Canada (Tamaki et al., 2023), and Germany (Pal et al., 2021).

ALS-FTD patient-associated point mutations in TDP-43 were investigated in eight studies *via* either overexpression or *via* point mutations engineered into the *TARDBP* gene (Fang et al., 2023, Gianini et al., 2020, Guerrero et al., 2019, Konopka et al., 2020, Lee and Rio, 2024, Marques et al., 2024, Mitra et al., 2025, Provasek et al., 2025, Pal et al., 2021). Three publications explored the role of *TARDBP* depletion or knockdown (Gianini et al., 2020, Marques et al., 2024, Mitra et al., 2019). Only one study investigated the overexpression role of wild-type *TDP-43* (Provasek et al., 2025). Finally, one study used samples from patients with sporadic rather than familial ALS-FTD (Tamaki et al, 2023).

Three primary methodologies were employed to assess the extent of DNA damage: immunostaining for γH2AX, which accumulates at sites of DSBs (Fang et al., 2023; Gianini et al., 2020; Guerrero et al., 2019; Konopka et al., 2020, Marques et al., 2024, Mitra et al., 2019, Mitra et al., 2025, Nogami et al., 2022, Pal et al., 2021, Provasek et al., 2025, Tamaki et al., 2023) (11/12 studies); Comet assays (Fang et al., 2023; Guerrero et al., 2019; Konopka et al., 2020; Mitra et al., 2019; Mitra et al., 2025; Provasek et al., 2025) (6/12 studies); and long amplicon-PCR (Guerrero et al., 2019; Mitra et al., 2019; Mitra et al., 2025) (3/12 studies) (Table 1). In addition, single studies used immunostaining for FANCD2 (Gianini et al, 2020), or 53BP1 (Pal et al, 2021), while TUNEL assay was used in one study (Mitra et al, 2025).

**Table 1:** Characteristics of the included studies focussing on TAR DNA-binding protein 43 (TDP-43).

Study	Country	Model system	Interventions	*TDP-43* mutations	DNA repair pathways	Method of detection
(A) Human and rodent cell line-based studies						
Gianini et al. (2020)	Italy/Spain	HeLa/SH-SY5Y	RNAi	75% KD/ G294V/ A382T	Fanconi Anemia pathway	H2AX and FANCD2 staining/(DRIP)-qPCR
Guerrero et al. (2019)	USA	SH-SY5Y	IR 3 Gy	Q331K	NHEJ	H2AX assay/Comet assay/LA-PCR/PLA assay
Fang et al. (2023)	USA	A549/HEK293/3C4	shRNA	KD	NHEJ/HR	H2AX assay/NHEJ, and HR assays
Mitra et al. (2019)	USA	SH-SY5Y	RNAi/ CRISPR-Cas9/ ETO/IR 6 Gy	30–75% KD/KO	NHEJ	H2AX assay/Comet assay/LA-PCR
Nogami et al. (2022)	Japan	U251	CLM	-	NHEJ (DNA-PK)	Live cell imaging
Mitra et al. (2025)	USA	SH-SY5Y	RNAi/ ETO	(mNLS)/KD	NHEJ	H2AX assay/MTT assay/Comet assay
Provasek et al. (2025)	USA	HEK293/SH-SY5Y	RNAi/CRISPR-Cas9 /GO	∼50% KD/ ΔNLS/ OE/ Q331K/ A315T/M337V	MMR	WB/Comet assay
Lee and Rio (2024)	USA	SH-SY5Y	-	KD/ N352S	-	Gene Ontology (GO) Term Analysis
Konopka et al. (2020)	Australia	NSC-34/Cortical neurons	ETO/H_2_O_2_/DNA-PK inhibitor	A315T/ Q331K	NHEJ	H2AX assay/NHEJ assay/Comet assay
(B) Brain organoid-based studies						
Tamaki et al. (2023)	Canada	Cerebral organoids	Injection of extracted protein from ALS patients	sALS containing pathogenic TDP-43	-	H2AX assay/WB/IHC
(C) iPSC-derived motor neurons from ALS patients						
Gianini et al. (2020)	Italy/Spain	LCLs	-	sALS/ A382T	Fanconi Anemia pathway	H2AX assay/co-IP/Flow cytometry
Fang et al. (2023)	USA	ALS patients	ETO/5-fluorouracil	M337V/ Q343R	NHEJ/HR	RNAi screening/comet assay/ survival assay/NHEJ/HR assays
Mitra et al. (2019)	USA	NPCs/MNs	RNAi/ETO	∼80% TDP-43 KD	NHEJ	H2AX assay/Co-IP/WB/PLA
Marques et al. (2024)	USA	fALS	-	KD/ TDP-43^+/G298S^	STING pathway	H2AX assay
Pal et al. (2021)	Germany	Spinal MNs	-	C9ORF72-ALS	-	H2AX and 53BP1 assay
Provasek et al. (2025)	USA	NPCs	-	KD/ Q331K	MMR	H2AX assay
Konopka et al. (2020)	Australia	Fibroblasts cells from ALS	fibroblasts derived from pre symptomatic and ALS patients	M337 mutation	NHEJ	H2AX assay/ICC/IB
(D) Rodent studies						
Mitra et al. (2025)	USA	TDP-43 KI mice	CRISPR/Cas9	KI (h*TARDBP* NLS)	NHEJ	H2AX assay /TUNEL assay/LA-PCR/ PLA assay
Provasek et al. (2025)	USA	NPCs/MNs	CRISPR-FLeX	OE (murine) TDP-43/ΔNLS	MMR	H2AX assay/WB
Konopka et al. (2020)	Australia	tgTDP-43 rNLS mice	-	cytoplasmic hTDP-43 with NLS	NHEJ	H2AX assay/IHC/ WB
(E) Post-mortem brain and spinal cord tissue from ALS patients						
Guerrero et al. (2019)	USA	SC tissue	-	Q331K mutation	Fanconi Anemia pathway	WB/ IHC/LA-PCR
Fang et al. (2023)	USA	Precentral gyrus Frontoinsular cortex	-	FTLD-TDP (C9ORF72 and sFTD-ALS)	NHEJ/HR	H2AX assay
Mitra et al. (2019)	USA	SC tissue (cervical region)	-	sALS cases (veterans and Chamorro)	NHEJ	IHC/IB/LA-PCR/TUNEL assay
Marques et al. (2024)	USA	Motor cortex and SC tissues	-	A315T and sALS	STING pathway	H2AX assay
Provasek et al. (2025)	USA	CNS tissues	-	Guamanian ALS patients	MMR	WB/RT-PCR

Genes and proteins: TDP-43, TAR DNA-binding protein 43; TARTDP, gene encoding TDP-43; FUS, fused in sarcoma protein; C9ORF72, chromosome 9 open reading frame 72. Disease classification: sALS, sporadic amyotrophic lateral sclerosis; fALS, familial amyotrophic lateral sclerosis, FTLD-TDP, frontotemporal lobar degeneration with TDP-43 pathology. Genetic tools and models: CRISPR/Cas9, clustered regularly interspaced short palindromic repeats/CRISPR-associated protein 9; RNAi, RNA interference; shRNA, short hairpin RNA; KI, knock-in; OE, overexpression; KD, knockdown; KO, knockout; tg, transgenic; FLeX, flip-excision system; rNLS, reduced or mutated nuclear localization signal. Experimental methods: IF, immunofluorescence; IHC, immunohistochemistry; WB, western blot; IB, immunoblotting; IP, immunoprecipitation; Co-IP, co-immunoprecipitation; ICC, immunocytochemistry; DRIP-qPCR, DNA–RNA immunoprecipitation followed by quantitative PCR; ChIP-seq, chromatin immunoprecipitation followed by sequencing; RT-PCR, reverse transcription polymerase chain reaction; Comet assay, single-cell gel electrophoresis for DNA damage; LA-PCR, long-amplicon PCR; PLA assay, proximity ligation assay; TUNEL assay, terminal deoxynucleotidyl transferase dUTP nick end labeling (apoptosis assay); MTT assay, colorimetric assay for cell viability; Gene Ontology (GO) analysis (bioinformatics method). Cell and tissue sources: iPSC, induced pluripotent stem cells; NPCs, neural progenitor cells; MNs, motor neurons; LCLs, lymphoblastoid cell lines; CNS, central nervous system; SC, spinal cord. DNA repair and mutagenesis assays: NHEJ, non-homologous end joining; HR, homologous recombination; EMS, ethyl methanesulfonate (mutagen). Chemical reagents and stressors: CLM, calicheamicin, ETO, etoposide (a topoisomerase II inhibitor); IR, ionizing radiation; H₂O₂, hydrogen peroxide (oxidative stress inducer), Glucose oxidase (GOx).

Outcomes were categorized as follows. The primary outcome aimed to evaluate the extent of DNA damage accumulation across the five models. Secondary outcomes examined dysfunction in both DDR and DNA repair mechanisms, focusing on the association between TDP-43 proteinopathy, increased DNA damage, DDR activation, and impaired repair processes.

### 3.4 Primary outcome: detection or accumulation of DNA damage in the CNS

The accumulation of DNA damage was investigated in each of the 12 studies. DSB were identified as the most prevalent form of DNA damage in neurons in 10 of the 12 studies (Fang et al., 2023; Gianini et al., 2020, Guerrero et al., 2019, Konopka et al., 2020, Mitra et al., 2019; Mitra et al., 2025; Nogami et al., 2022; Pal et al., 2021, Provasek et al., 2025, Tamaki et al., 2023) but other types of DNA damage were also identified: increased SSB (Provasek et al., 2025; Fang et al, 2023) and abnormal R-loop formation (Gianini et al, 2020), plus reduced DNA amplification (Gianini et al., 2020), elevated DNA mutation rates (Fang et al, 2023; Guerrero et al., 2019) and increased reactive oxygen species (ROS) levels (Guerrero et al, 2019). The methods used to identify these changes are described below to facilitate comparison between studies.

### 3.5 DNA damage quantification

Significant quantitative evidence of the accumulation of DNA damage was shown, most notably in the studies involving *TARDBP* knockdown or mutations linked to ALS-FTD. Mitra et al. (2019) demonstrated a 20-fold increase in DNA damage when TDP-43 was depleted in differentiated SH-SY5Y cells compared to the controls. Moreover, Fang et al. (2023) revealed a correlation between DNA damage and the aggregation of TDP-43 in ALS-FTD patient brain tissues. In the precentral gyrus of ALS brains, 62% of neurons with TDP-43 cytoplasmic inclusions also showed γH2AX foci, while the remaining 38% of the neurons retained normal TDP-43 (*p* = 0.0229) (Guerrero et al., 2019). Similarly, in the frontoinsular cortex of FTD patient brains, 35% of neurons with TDP-43 cytoplasmic inclusions also showed γH2AX foci, while only 21% of neurons with normal nuclear TDP-43 exhibited such damage (*p* < 0.001) (Fang et al, 2023). Mitra et al. (2025) used TUNEL analysis to detect DNA damage in the cerebral cortex and spinal cord of 12-month-old transgenic mice expressing mislocalized TDP-43 (MN-Tdp-43∆NLS) compared to sham control. The number of TUNEL-positive nuclei exhibited a 24.40 ± 3.54% increase in the cortex and a 31.10 ± 3.26% increase in the spinal cord compared to sham (cortex and spinal cord both *p* = 0.0001) (Table 2).

**Table 2:** Key findings associating dysfunction of TDP-43 with DNA damage or the DNA damage response.

Author/Year	DNA damage	DDR components affected	DNA repair
(A) Human and rodent cell line-based studies			
Gianini et al. (2020)	R-loops causing DSBs	ATM, H2AX, 53BP1, FANCD2	Significant increase in H2AX and FANCD2
Guerrero et al. (2019)	SSB/ DSB /reduction in DNA amplification	ATM, H2AX, 53BP1 (>4-6-fold increase in DDR factors)	Delayed in DSB repair after IR/ cytosolic accumulation of the XRCC4-DNA ligase 4 complex
Fang et al. (2023)	Increase in mutation rate/ DSBs	ATM	2-3-fold reduction in HR and NHEJ activity
Mitra et al. (2019)	Accumulation of DNA damage/ DSBs (20 fold)	ATM, H2AX, 53BP1	After ETO, increased TDP-43’s interaction with DSB repair proteins, but not with SSB-associated
Nogami et al. (2022)	DSBs/ Induced rapid movement of TDP-43 to SGs after CLM	-	DNA-PK
Mitra et al. (2025)	DSBs/ Reduction in cell viability/ increase in genomic instability	ATM, H2AX, 53BP1	2.47-fold reduction in DSB repair kinetics
Provasek et al. (2025)	Increase in DNA damage	MMR factors/ ATM/H2AX	significant changes in DNA repair proteins expression
Lee and Rio (2024)	No results available	No results available	Affects splicing of 593 common genes linked to DNA repair
Konopka et al. (2020)	Increase in DNA damage after ETO	H2AX, 53BP1, DNA-PKcs	Smaller H2AX foci in WT cells (*p* < 0.001)/KD cells prevented pH2AX following ETO/ WT, A315T and Q331K colocalized with 53BP1 after ETO/ affected cNHEJ, but not aNHEJ/ TDP-43’s DNA repair function depends on DNA-PK
(B) Brain organoid-based studies			
Tamaki et al. (2023)	DSBs	Increase in H2AX with some colocalized with cytoplasmic pTDP-43 aggregates	No results available
(C) iPSC-derived motor neurons from ALS patients			
Gianini et al. (2020)	R-loops causing DSBs	ATM, H2AX, 53BP1, FANCD2	Significant increase in H2AX and FANCD2
Fang et al. (2023)	Increase DNA mutation rate/ reduction in survival following ETO and 5-fluorouracil in mutant neurons	ATM	~2-fold and 2–3 fold reduction in NHEJ and HR in mutant neurons
Mitra et al. (2019)	Increased DNA damage and apoptosis	ATM, H2AX, 53BP1	WT: rapidly interaction with DDR factors (γH2AX, pATM)/ KD: 4–6 fold accumulation of DSB factors/ a 16-10-fold increase in NHEJ factors, but not SSB repair factors after ETO
Marques et al. (2024)	Significant DNA damage/ STING pathway activation	H2AX	No results available
Pal et al. (2021)	Increased DNA damage/DSBs	H2AX, 53BP1	No results available
Provasek et al. (2025)	SSBs/ DSBs	MMR factors/ ATM/H2AX	No results available
Konopka et al. (2020)	Increase DNA damage (53BP1 foci) in pre-symptomatic with further increase in M337V ALS cells	H2AX, 53BP1, DNA-PKcs	Impaired NHEJ repair/ increase 53BP1 and γH2AX foci
(D) Rodent studies			
Mitra et al. (2025)	DSBs/ increase in genomic instability/ significantly higher γH2AX foci and senescence markers in both whole-body and motor neuron	ATM, H2AX, 53BP1	Mislocalized TDP-43 trapped NHEJ factors
Provasek et al. (2025)	No results available	Both models showed increase in MMR factors	No results available
Konopka et al. (2020)	DNA damage in presymptomatic stage/γH2AX foci were correlated with cytoplasmic TDP-43	H2AX, 53BP1, DNA-PKcs	No results available
(E) Post-mortem brain and spinal cord tissue from ALS patients			
Guerrero et al. (2019)	DNA damage instability	ATM, H2AX, 53BP1	No results available
Fang et al. (2023)	Significant increase in DSBs	ATM, H2AX	Significant increase in γH2AX foci in both tissues (a and b)
Mitra et al. (2019)	Highly increased DNA damage/ decreased in DNA integrity/increased TUNEL staining	ATM, H2AX, 53BP1	Significant reduction in DSB ligation efficiency
Marques et al. (2024)	STING accumulates in layer V cortical pyramidal neurons	No results available	No results available
Provasek et al. (2025)	No results available	Higher level of MLH1 protein in the insoluble protein fraction in CNS	Accumulation of MLH1 pre-mRNA levels in the ALS CNS samples

Notably, evidence for early accumulation of DSB was consistently documented across various human and mouse models studying different TDP-43 mutations and tissue types. Konopka et al. (2020) reported a significant elevation in DNA damage in human dermal fibroblasts derived from a presymptomatic TDP-43 M337V carrier using immunostaining for 53BP1, a marker that accumulates at sites of DSBs, compared to controls (*p* < 0.05). Moreover, in cortical neurons from early-stage rNLS mice expressing TDP-43, cytoplasmic mislocalization of TDP-43 was found to correlate with an increased γH2AX signal (*p* < 0.05) (Konopka et al, 2020) (Table 3).

**Table 3:** Summary of studies investigating the impact of TDP-43 proteinopathy on DNA repair mechanisms and associated cellular outcomes.

Study	TDP-43 proteinopathy	Impact on DNA repair	Cellular outcome	Statistical evidence
Gianini et al. (2020)	KD/A382T/G294V	Increased R-loop/ FA impairment	DNA damage/genome instability	Significant increase (*p*-values not provided)
Guerrero et al. (2019)	Q331K	Impaired NHEJ/ reduced XRCC4-DNA ligase 4 recruitment	DNA damage/ neuronal apoptosis	>4-6-fold increase in DDR factors
Fang et al. (2023)	M337V/Q343R	NHEJ and HR reduction	Increased genomic instability	2-3-fold decrease in repair activity
Mitra et al. (2019)	KD	Impaired NHEJ	DSBs	20-fold increase in DSBs
Mitra et al. (2025)	TDP-43 NLS variant	Impaired NHEJ/ sequestration of repair proteins	Increased DNA damage/ neuronal senescence	2.47-fold increase in unrepaired DSBs (*p* < 0.0001)
Provasek et al. (2025)	KD/OE/Q331K/ A315T/ M333V	Altered MMR gene expression	Increased DNA damage	No results available
Lee and Rio (2024)	N352S (public datasets)	Altered splicing of DNA repair genes	Potential impact on DNA repair	No results available
Konopka et al. (2020)	A315T/Q331K	Impaired NHEJ	Accumulation of DNA damage	Change in area and volume of H2AX foci *p* < 0.001

TDP-43, TAR DNA-binding protein 43; KD, knockdown; OE, overexpression; NHEJ, non-homologous end joining; HR, homologous recombination; MMR, mismatch repair; DSBs, DNA double-strand breaks; FA, Fanconi anemia pathway; XRCC4, X-ray repair cross-complementing protein 4; H2AX, histone H2A variant X (γH2AX is its phosphorylated form and a marker of DSBs); DDR, DNA damage response.

Marques et al. (2024) reported an increase in the γH2AX signal indicating DSB in iPSC-derived neurons following TDP-43 depletion, compared to scrambled controls (*p* < 0.001). The same study also reported activation of the STING pathway, a mediator of innate immune signaling in response to DNA damage, in glial cells and selectively in layer V cortical neurons, but not in layer ll/lll neurons from ALS patients with a TDP-43 A315T mutation, when compared to controls (*p* < 0.01).

### 3.6 Behavioral studies in animal models

Two studies examined the accumulation of DNA *in vivo* using mouse models (Konopka et al., 2020; Mitra et al., 2025), and provided evidence indicating a correlation between accumulating DNA damage and both functional motor impairment and neurodegeneration. Mitra et al. (2025) demonstrated notable motor deficits in the MN-Tdp-43∆NLS mouse using the rotarod (8–30 rpm), irregular hindlimb movements during tail suspension tests, and changes in gait characteristics, such as a smaller paw area and shorter stride length. Similarly, Konopka et al. (2020) demonstrated progressive neurodegeneration and motor impairments accompanied by an increase in γH2AX foci – indicating a rise in DSB – within the cortex of transgenic mice expressing cytoplasmic human TDP-43.

These consistent findings of accumulating DNA damage across the multiple *in vitro* and *in vivo* models of TDP-43-associated ALS-FTD we reviewed, are an indicator that DNA damage is an important factor in the pathophysiology of ALS-FTD. This view is further strengthened when the multiple instances from post mortem patient brain tissue of correlations between levels of DSB and TDP-43 inclusions are factored in, plus the extremely high incidence of TDP-43 inclusions found in ALS-FTD patient tissues.

### 3.7 Secondary outcomes

#### 3.7.1 The DNA damage response

A common theme emerging from our review was that DNA damage accumulates across the various models and studies. It is therefore, to be expected that components of the DNA damage response (DDR) should also be raised as cells recognize and attempt to repair the damage. As predicted, increased levels of DDR were reported in 11/12 studies (Fang et al., 2023; Gianini et al., 2020; Guerrero et al., 2019; Konopka et al., 2020; Marques et al., 2024; Mitra et al., 2019; Mitra et al., 2025; Nogami et al., 2022; Pal et al., 2021; Provasek et al., 2025; Tamaki et al., 2023).

There are multiple ways of quantifying the DDR. One is to visualize the repair response mounted to damage using antibodies to proteins that accumulate at the sites of damage: γH2AX and 53BP1 both accumulate at DSB as early components of the response and hence, both are commonly used as markers to visualize and quantify breaks. In our review, 9 of 12 studies used γH2AX to quantify DSB (Fang et al., 2023; Gianini et al., 2020; Guerrero et al., 2019; Konopka et al., 2020; Marques et al., 2024; Mitra et al., 2019; Mitra et al., 2025; Nogami et al., 2022; Pal et al., 2021) and 6 employed 53BP1 (Gianini et al., 2020; Guerrero et al., 2019; Konopka et al., 2020; Mitra et al., 2019; Mitra et al., 2025; Pal et al., 2021). ATM, the central regulator of the DDR, also accumulates at DSB and was used in 6 studies (Fang et al., 2023; Gianini et al., 2020; Guerrero et al., 2019; Mitra et al., 2019; Mitra et al., 2025; Provasek et al., 2025). Other DDR factors involved in DSB repair, were also quantified: DNA-PKcs (Konopka et al, 2020), and DNA-PK (Nogami et al, 2022) plus FANCD2, which is used to quantify replication stress (Gianini et al, 2020).

The level of activation of the DDR exhibited comparable patterns across the included studies. The study conducted by Guerrero et al. (2019) reported an increase by 4-6-fold in DDR factors in SH-SY5Y neuroblastoma cells harboring the Q331K mutation: specifically, γH2AX, p-ATM and p-53BP1 levels were raised, indicating an increase in DSB. Similarly, mice expressing the human *TARDBP* gene with a mutated nuclear localization sequence (NLS) that leads to accumulation of the TDP-43 protein in the cytosol, exhibited an increase in the γH2AX signal by 4-fold compared to controls (Mitra et al, 2025). In addition to changes in the levels of DDR components, the size of γH2AX foci was also affected by several TDP-43 mutations. The A315T and Q331K mutations were associated with an increase in both area and volume of the γH2AX foci in cells when compared to controls (*p* = 0.05 and 0.0001, respectively) (Konopka et al, 2020). An increase in foci size likely indicates a combination of increased damage and inefficient repair.

#### 3.7.2 Impairment in DNA repair processes

Post-mitotic neurons are particularly reliant on non-homologous end-joining (NHEJ) for the repair of DSBs as there is no sister chromatid available to act as a repair template for homologous recombination. NHEJ is error-prone and hence, neurons may be susceptible to genome instability in the event of increased DNA damage, which may be exacerbated by transcriptional activity. Due to this reliance of neurons on NHEJ, in the TDP-43 studies that looked for signs of impaired DNA damage repair, NHEJ was the focus [7/12 studies (Fang et al., 2023; Guerrero et al., 2019; Mitra et al., 2019; Mitra et al., 2025, Nogami et al., 2022, Konopka et al., 2020, Tamaki et al., 2023)]. Homologous recombination (HR) (Fang et al, 2023), mismatch repair (MMR) (Provasek et al., 2025) and the Fanconi Anemia pathway that repairs interstrand crosslinks (Gianini et al, 2020) were each investigated in only a single study.

Consistent with the increase in markers of DNA damage, several models reported a significant impairment in DNA repair efficiency. Fang et al. (2023) saw a 2–3 fold reduction in both NHEJ and HR rate in cells where *TARDBP* expression had been knocked down, plus similar correlative results were obtained from ALS patient iPSC-derived neurons carrying the TDP-43 mutations, M337V or Q343R, when compared to controls (*p* < 0.05). Similarly, Mitra et al. (2025) reported a 2.5-fold increase (*p* < 0.0001) in the accumulation of unrepaired DSB in TDP-43 mNLS expression neuronal cells. Guerrero et al. (2019) reported that cells harboring the Q331K mutation showed reduced DNA strand break sealing activity, attributed to impaired nuclear translocation of the XRCC4-DNA ligase 4 complex, compared to control cells (*p* < 0.0001). The outcomes were consistent across the different studies and methods: DNA damage repair is likely to impaired by a reduction in levels of, or mutation in, TDP-43, leading to the potential for genome instability.

#### 3.7.3 TDP-43 proteinopathy and DNA repair functions

The link between TDP-43 proteinopathy and the DNA damage response was a particular focus of many of the studies reviewed. Nine of twelve studies looked directly at this area (Fang et al., 2023; Gianini et al., 2020; Guerrero et al., 2019; Konopka et al., 2020; Lee and Rio, 2024; Marques et al., 2024, Mitra et al., 2019, Mitra et al., 2025, Provasek et al., 2025). Table 3 summarizes the TDP-43 models investigated, the effects on DNA repair and outcomes for cells. The data reveal a correlation between TDP-43 status, the extent of DNA damage, and the efficacy of DNA repair processes.

Two main approaches were used. The majority of studies (6/12) used point mutations identified in familial or sporadic cases of ALS or ALS-FTD to ask questions about how mutated TDP-43 correlates with an increase in DNA damage and activation of DDR (Fang et al., 2023, Gianini et al., 2020, Guerrero et al., 2019, Konopka et al., 2020, Marques et al., 2024, Provasek et al., 2025). A subset, plus additional studies, also manipulated *TDP-43* expression, via knockdown or overexpression (Gianini et al., 2020; Marques et al., 2024; Mitra et al., 2019; Mitra et al., 2025; Provasek et al., 2025). Finally, one study used exclusively *in silico* techniques to study a point mutant: TDP43-N352S (Lee and Rio, 2024).

Seven of the studies demonstrated a direct reduction in DNA repair efficiency across various models of TDP-43 proteinopathy (Fang et al., 2023; Gianini et al., 2020; Guerrero et al., 2019; Konopka et al., 2020; Mitra et al., 2019, Mitra et al., 2025, Tamaki et al., 2023). This was complemented by two studies highlighted an additional indirect transcriptional mechanism at play: Lee and Rio (2024) reported that the TDP-43 N352S mutation in SH-SY5Y cells altered the splicing of genes related to DNA damage and Provasek et al. (2025) reported that TDP-43 depletion significantly affected the expression of key MMR genes in HEK293 cells, compared to cells transfected with a scrambled control vector (*p* < 0.0001).

Importantly, similar alterations in MMR gene expressions to those seen in HEK293 cells were observed in two *in vivo* ALS mouse models expressing nuclear-excluded TDP43-ΔNLS, when compared to controls (*p* < 0.05) (Provasek et al., 2025). Moreover, levels of the Mlh1, Msh2, and Msh3 MMR proteins were elevated in the insoluble protein fraction extracted from the cortical and spinal cord tissue of patients with sporadic ALS, relative to controls (*p* < 0.05) (Provasek et al., 2025).

Reviewing the findings across the different models, system and approaches, there is evidence to support a correlation between TDP-43 proteinopathy and dysregulation of MMR.

## 4 Discussion

This systematic review and narrative synthesis of 12 studies included both *in vitro* and *in vivo* models that all investigated the connection between TDP-43 proteinopathy in ALS-FTD and DNA damage and the DNA damage response. From the results obtained, several key conclusions can be drawn. Firstly, accumulation of DSB is a common feature resulting from disruption to TDP-43 homeostasis, be that through depletion, overexpression or introduction of disease-related point mutations, or in patient-derived cells. Secondly, dysregulation of NHEJ is a common feature, but wider dysregulation of DNA repair pathways is also seen, with likely implications for genome stability. Thirdly, the effects of TDP-43 proteinopathy on DNA damage and repair are similar across a broad range of models, from *in vitro* to *in vivo* to *post-mortem* patient tissue, which allows us to be confident in the conclusions, albeit that only 9 or 12 studies investigated proteinopathy directly.

### 4.1 Impact of TDP-43 disruption on DNA repair pathways

The studies reviewed collectively demonstrated that the NHEJ pathway was the primary DNA repair mechanism disrupted by TDP-43 proteinopathy (Fang et al., 2023; Guerrero et al., 2019; Konopka et al., 2020; Mitra et al., 2019; Mitra et al., 2025; Nogami et al., 2022; Tamaki et al., 2023). This disruption manifests in several ways, including impairing the recruitment of key NHEJ factors such as the XRCC4-LIG4 complex (Guerrero et al., 2019, Konopka et al., 2020, Mitra et al., 2025). This disruption to NHEJ is a probable cause of the accumulating, unrepaired DSB and is likely to have a greater impact on non-proliferating cells, such as neurons, since HR requires a sister chromatid to act as a template and can therefore operate only after DNA replication. Other DNA repair pathways were also affected by TDP-43 proteinopathy included HR (Fang et al, 2023), mismatch repair (Provasek et al., 2025), and the Fanconi Anemia pathway (Gianini et al, 2020). This indicates a broader influence of TDP-43 on the maintenance of genomic stability.

### 4.2 Clinical implications

Accumulation of DNA damage or impaired DNA repair was a consistent observation in patient-derived neurons and in postmortem samples (Provasek et al., 2025). The studies using animal models also demonstrated a correlation between DNA damage and motor dysfunction *in vivo* (Konopka et al., 2020, Mitra et al., 2025). The inference, therefore, is that loss of TDP43 or TDP-43 proteinopathy resulting from mutation triggers dysregulation to NHEJ, MMR and other DNA repair pathways, and that the resultant genome instability is fundamental to the disease process. If this is true, and given that TDP-43 proteinopathy is seen in a very high proportion of cases of ALS-FTD, then disrupted DNA repair is likely to be a key contributor to the pathophysiology of ALS-FTD. Methods of reducing DNA damage accumulation or enhancing repair pathways may be effective therapeutic options.

One such approach is highlighted by a recent paper by Modafferi et al. (2025) that was published after completion of the screen and data extraction for this review. Modafferi et al. (2025) identified a rise in nuclear DSB detected by an increase in γH2AX or 53BP1 foci in HeLa cells with cytosolic aggregates of either TDP-43 or FUS proteins. Treatment of these cells with the drug, Enoxacin, reduced the level of DNA damage. Enoxacin is an agonist of the miRNA processing enzyme, DICER, that plays a key role in miRNA processing – miRNA processing is essential for motor neuron survival and there is a global reduction of miRNA levels in ALS (Emde et al., 2015). Interestingly, DICER is recruited to DSB and is crucial for full activation of the DDR (Gioia et al., 2019). Modafferi et al. (2025) were able to show that enoxacin treatment is able to accelerate NHEJ in HeLa cells to reduce DNA damage (Modafferi et al., 2025). Enoxacin and has shown promise reducing the motor pathology in TDP-43 A315T and SOD1 G93A mouse models (Emde et al., 2015) and is currently in early phase trials is the treatment of ALS patients with the fluoroquinolone, enoxacin (Clinical trial: NCT04840823).

Given TDP-43 aggregates are almost ubiquitous in ALS patients, and also extremely common in ALS-FTD patients, indirect therapies, such as Enoxacin, that target the downstream consequences are a promising approach for the 90% of cases that are sporadic and without a clear genetic cause. Moreover, TDP-43 aggregates are commonly found in other neurodegenerative diseases, including in Alzheimer’s disease, so similar dysregulation of DNA damage repair as discussed here may also contribute to disease. Hence, if general therapies targeting the DDR are effective for ALS-FTD patients, they may also be effective more generally for neurodegeneration.

### 4.3 Methodological considerations

Immunostaining for γH2AX and biochemical Comet assays were the most frequently employed methods for assessing DNA damage levels in the studies reviewed. Given the widespread use of these techniques, the observed variations in result reporting underscore the necessity for standardized measurement protocols in future research. Different studies differed in their approach to reporting: some used qualitative descriptions, while others provided exact fold changes and statistical *p*-values. This inconsistent reporting makes it harder to evaluate results across studies. Immunostaining or biochemical methods such as Comet assay methods offer only a snapshot assessment of DNA damage levels and new methods that allow for longitudinal measurements of DNA damage would add valuable information on the kinetics of DNA damage accumulation.

### 4.4 Risk of bias

The TDP-43 models reviewed here comprised both *in vitro* and *in vivo* methods. The assessment of *in vitro* studies reviewed indicated a low risk of bias, with most studies meeting the criteria for high methodological quality across domains. Hence the conclusions can be considered reliable. Conversely, the vertebrate single *in vivo* study (Nogami et al., 2022) showed a high risk of bias in four SYRCLE domains: baseline characteristics, randomization, allocation concealment and outcome assessment. Given that high risk occurs in multiple domains, the strength of the conclusions emerging from the study should be considered as potentially unreliable.

### 4.5 Further limitations of the study

The small number of studies meeting the inclusion criteria in this review and narrative synthesis may restrict the generalizability of the findings. However, the findings herein can be viewed in a broader context when considered alongside those of the two related studies that systematically reviewed data from pre-clinical models of ALS-FTD associated with two further genes: C9orf72 (Almalki et al., 2025a) and FUS (Almalki et al., 2025b). The concentration of studies from the USA may be considered a limitation, as may the restriction of the search to publications only in English.

## 5 Conclusion

This systematic review and narrative synthesis investigated the connection between DNA damage and TDP-43 dysfunction and proteinopathy. Our findings indicate a strong association between TDP-43 disruption and increased DNA damage across various different models and across different studies. This heightened DNA damage may result from disrupting a scaffolding function of TDP-43 for key components of the DNA repair pathways, rather than from direct induction of DNA damage. Elevated DDR factors suggest that DNA damage continues to be detected in these cases, but that repair efficiency is compromised, potentially due to impaired nuclear transport caused by TDP-43 proteinopathy. The potential for conducting meta-analyses to aggregate results was prevented by significant variability in study design and outcome reporting and there is a need for standardized methodologies to be adopted for assessing and reporting DNA damage and the DDR. Finally, two important limitations must be considered when interpreting the results: firstly, this systematic review and narrative synthesis only covered eight TDP-43 mutations but nearly 40 have been linked to forms of ALS-FTD and, secondly, some mutations were only investigated in a single study.

## Data Availability

The original contributions presented in the study are included in the article/supplementary material, further inquiries can be directed to the corresponding author.
